# Caught on the Fringes of Life: Mothers’ Lived Experiences of Initial Breastfeeding Complications

**DOI:** 10.1177/10497323211002484

**Published:** 2021-04-07

**Authors:** Lise-Lott Rydström, Azar Tavallali, Eva Sundborg, Anita Berlin, Albertine Ranheim

**Affiliations:** 1 Karolinska Institute, Stockholm, Sweden

**Keywords:** breastfeeding complications, caring encounters, lifeworld approach, qualitative, reflective lifeworld research, Sweden

## Abstract

Becoming a parent changes one’s life, and existential questions arise. Time and being oscillate between joy and powerlessness, vulnerability, and self-confidence, between harmony and unpreparedness. Breastfeeding, one of the first skills new mothers try to master, can be joyful and painful. The aim of this study was to develop a deeper understanding of the phenomenon of initial breastfeeding complications as they are lived and experienced by mothers and how these mothers can be supported. Twelve mothers were interviewed, and a phenomenological lifeworld approach was used. Nourishing an infant and having initial breastfeeding complications can be understood by the essence and its constituents. Expectations are fulfilled, and expectations come to naught when complications are experienced such as wavering between powerlessness and joy and finding solutions through resistance. The results suggest that lifeworld-led caring may lead to deepened acknowledgment and the possibility for mothers to feel supported in their extreme situations.

## Background

Becoming a parent is a life-changing transition associated with great wonder and joy, but also with feelings of insecurity, insufficiency, and vulnerability as well as with feelings of being unprepared for the new role and situation (Deave et al., 2008). Studies have shown that concomitant with this new role are step-by-step changes whereby parents learn to cope with new responsibilities and the fear of disrupting the life of the growing child (Cast, 2004; Lawrence et al., 2007). A challenging but important facet associated with early-stage parenthood is the responsibility for providing the infant with optimal living conditions and nourishment (Lessen & Kavanagh, 2015). Breastfeeding is generally considered the best way of feeding an infant (Lessen & Kavanagh, 2015; World Health Organization [WHO] 2011, 2020), and it is well known that breastfeeding is associated with lower mortality during the first 6 months of life.

Despite knowledge about the benefits of breastfeeding, the frequency of breastfeeding has decreased in Sweden and other developed countries such as the United Kingdom, the United States, and Norway (Brockway & Venturato, 2016; Victora et al., 2016). The decision to breastfeed or not to is based on several circumstances. Factors influencing a mother’s decision regarding breastfeeding her child are based on contextual and existential matters and the support that the mother has (Hauck et al., 2016).

Breastfeeding is consequently one of the first skills many new mothers try to master, but despite the knowledge of the advantageous impacts of breastfeeding, mothers often stop breastfeeding for different reasons (Almqvist-Tangen et al., 2012; Schafer et al., 2016; Williamson et al., 2012). Such reasons can include different complications when breastfeeding, such as poor weight gain (Almqvist-Tangen et al., 2012), poor suckling ability (Almqvist-Tangen et al., 2012; Schafer et al., 2016), and difficulties finding a comfortable position for breastfeeding (Williamson et al., 2012).

Initial complications related to infant breastfeeding appear to leave mothers in a vulnerable and exposed position in which expectations and responsibility are intermingled, and this might lead to a measurable risk for early termination of breastfeeding (Larsen-Schilling & Kronborg, 2013). It is known that when breastfeeding support is offered to mothers, the duration and exclusivity of breastfeeding is increased. Strategies and care that rely mainly on face-to-face support are more likely to succeed with mothers practicing exclusive breastfeeding (Renfrew et al., 2012), and this indicates that there is a need to give voice to mothers’ stories and lived experiences. Interrelated dimensions of breastfeeding as embodied knowledge of the interdependence between the woman (mother) and her baby are expressed as the body knowing at a prelogical, prearticulated level (Ryan et al., 2011). Failing to recognize the importance of this level of discourse may lead to missing out of needs in the caring of mother and child.

We need to know more about what kind of supportive care is needed in individual situations and how to provide that knowledge to health care personnel. It is important to avoid early breastfeeding termination and to secure breastfeeding for infants in line with the WHO (2011, 2020) recommendations. The aim of this study was to develop a deeper understanding of the phenomenon of initial breastfeeding complications as they are lived and experienced by mothers and how these mothers can be supported.

## Design

To develop a deeper understanding of mothers’ experiences, a phenomenological approach and design with an open wondering and nonjudging attitude was used (H. Dahlberg et al., 2019; van Manen, 2014). We specifically wanted to explore how a phenomenological lifeworld approach (K. Dahlberg et al., 2008) can help broaden the understanding of the phenomenon of breastfeeding infants, focusing on mothers who had initial breastfeeding complications. With such a lifeworld approach, we also wanted to explore what skills and knowledge health care personnel need in their encounters with mothers experiencing such complications.

## Approach

A key aspect of a phenomenological approach is to inquire into the “whatness,” that is, how the phenomenon appears and presents itself as an experience that is recognizable. This entails an investigation into intersubjective understanding, that is, the lived significance of an experience itself and how this experience is shared, spoken of, and communicated with others (Adams & van Manen, 2017). A phenomenological approach means embracing a lifeworld perspective (H. Dahlberg et al., 2019; K. Dahlberg et al., 2008; Todres et al., 2006). Thus, the lifeworld is a point of departure from which we understand the world, our experiences, and our existence. It is consequently a theory that encompasses the entirety of human existence. Thus, it is from within the lifeworld that we understand the world, and it is the lifeworld that shapes our experiences and our existence. This “world” is highly relational, interpersonal, and intersubjective and affects the way we see ourselves and each other and how we understand existential phenomena such as health, living conditions, environment, illness, learning, and caring. In short, the lifeworld means a view of our relationship with our world and everything that comes with it (H. Dahlberg et al., 2019).

The lifeworld concept, along with its philosophical foundations, originates with the German philosopher Husserl (1980) and pays attention to the conditions that are taken for granted in the world we inhabit and live in. Having this in mind, throughout the research process, it becomes essential to not make something definite when it is indefinite (H. Dahlberg & Dahlberg, 2003). “Being bridling” (as in bridling a horse), is thus an ongoing reflection on the phenomenon under study throughout the process of understanding the phenomenon in a new way. It is thus different from just focusing upon the preunderstanding and consequently, it is not something that is performed just once. The researchers consistently relate to the demands bridling from the planning phase of the study, all through the data gathering and analysis, and in reporting the study (K. Dahlberg et al., 2008). Such reflective approach is essential for the sake of scientific quality.

In the context of health care, a lifeworld perspective means encountering the patient’s lifeworld and listening to the patients and their perceptions of the study object, as well as respecting the patient as a specialist on him or herself. This is foundational for an individual caring encounter (H. Dahlberg et al., 2019; Todres et al., 2006).

## Study Setting

The present study was carried out in Sweden where the health care offered to expectant and new parents is similar for all parents when it comes to antenatal clinics, delivery and maternity wards, and child health care service units. Hospital maternity wards are responsible for mothers and children up to 1 week after birth, and the nationwide child health care system provides regular checkups for children aged 0 to 5 years (The National Handbook of Child Health Services, 2019; Regions Stockholm, 2020). The entire health care chain serves parents and their children with general interventions in which midwives and child health care nurses are both involved. Breastfeeding information and support are one of the important interventions (The National Handbook of Child Health Services, 2019; Region Stockholm, 2020). In addition to government-financed support and, in the big cities, private practice midwives are available, offering breastfeeding consultancy by telephone contact or clinical visits. To what extent new parents make use of this service is unknown.

A majority of expectant and new parents in Sweden have an ambition to initiate breastfeeding, and statistics reveal that 95% of infants are exclusively breastfed 1 week after birth (National Board of Health and Welfare, 2017). In line with the WHO (2011, 2020) recommendations, Swedish national public health awareness authorities and programs emphasize the importance of breastfeeding the infant up to the age of 6 months (National Board of Health and Welfare, 2017; The National Handbook of Child Health Services, 2019; Region Stockholm, 2020; Swedish Food Agency, 2012). Despite these recommendations, the proportion of women who choose to breastfeed has varied and declined over the years. From 1995 to 2004, approximately 72% of all infants were still breastfed at the age of 6 months, but this has declined to 63% today (National Board of Health and Welfare, 2017; Region Stockholm, 2019). In Sweden, there are no clear confirmed explanations for what makes women choose to breastfeed or not. However, some possible explanations have been discussed, such as the negative impact of the brief maternity ward stay after delivery, which in most cases is only 2 days. This may contribute to parents being unprepared for breastfeeding challenges, lacking practical guidance, and not knowing where to turn for help (Chasson & Taubman-Ben-Ari, 2020).

Even though nearly all parents and their children visit child health care on a regular basis where breastfeeding support is on the agenda, breastfeeding has steadily declined in Sweden (National Board of Health and Welfare, 2017; Region Stockholm, 2019). To reverse this negative trend, one child health care organization in Stockholm took the initiative to establish a specific clinic focusing on supplementary, early, and qualified breastfeeding guidance to parents with initial breastfeeding complications. Mothers in need could find information on the web or could receive information from health care personnel in antenatal care, maternity wards, and child health services.

## Ethical Reflections

The project was approved by the regional ethical review board in Stockholm (Dnr: 2018/1230-31). In line with the declaration of research (World Medical Association, 2018), all participants received an information letter outlining the purpose of the study, the procedure, and their right to withdraw at any time. All participants gave their written approval in the main study. In addition, prior to the interviews, an additional verbal approval was confirmed over the phone and participants received information regarding the intended purpose and use of the interview study and their rights as participants were repeated. Procedures to ensure confidentiality were taken such as keeping all data anonymous, locked, and coded in accordance with current rules from the ethical board. It is not possible to trace particular informants. The informants will be given the possibility to view the results of their participation when the written article is published, and if so, they will be offered an oral walkthrough with the research team.

## Participants and Sampling

Participating mothers in the present study were recruited from an evaluative study (*n* = 161) after visiting a primary health care clinic for mothers with breastfeeding complications in Sweden, led by child health care nurses. In the questionnaire, they were asked whether they consented to be contacted for a subsequent interview study. Twenty mothers answered “yes,” and of these, a total of 15 mothers were strategically selected. To ensure breadth in the data, participants were selected with a variation of mothers’ age, number of children, socioeconomic status, and type of complication. When contacted by two of the researchers, 15 mothers accepted participation, but three mothers opted to cancel the interviews before they took place. Thus, in the end, 12 of the mothers were interviewed on one occasion.

The participating mothers were 28 to 39 years old (with *M* age of 31.5 years). Seven of the participants were first-time mothers, four had had their second child, and one had her third child. Eleven of the participating mothers lived in a relationship and one was a single mother. All participating mothers stated that they had decided to breastfeed already during their pregnancy. The participating mothers visited the clinic 1 or 2 times, due to initial breastfeeding complications.

### Data Collection

The interviews were conducted 4 to 6 months after the mother had visited the clinic for the first time. The interviews were performed during January to May 2019, at the time and place chosen by the participants. Eleven interviews took place in the participant’s homes and one in the participant’s workplace.

A relaxed conversational immediacy was strived for, to deepen the dialog in the interviews (K. Dahlberg et al., 2008; Kvale & Brinkman, 2014). A central factor in reflective lifeworld research is an attitude to discover what stands before us while being open to the meaning of an experience.

Participants were asked two initial questions: *How was it like to become a mother?* and *How was it like to breastfeed your child?* From these questions, the participants were encouraged to describe their experiences in their own words and to narrate issues that were important to them in relation to breastfeeding, motherhood, and their encounters with health care. To get in-depth answers, follow-up questions such as *Can you tell me more?* and *Can you give any examples?* were asked.

The participants were eager to talk about their experiences, and the interviews were rich in significance even when shorter in time. Often their child/children were present as well, and as such, the participants got substantial in their narratives. When asked about the meaning of their breastfeeding complications, the mothers gave nuanced and diversified pictures of their situation All five authors participated in conducting the interviews, and two were present at every interview after getting the informants specific informed consent on this. The interviews lasted 21 to 55 minutes, ranged in length from 3,170 to 9,382 words (*M*: 5,662 words), and they were tape-recorded and transcribed verbatim.

## Analysis Process

The interview data were analyzed manually without any use of analytic software and followed a phenomenological and reflective lifeworld research approach as presented by K. Dahlberg et al. (2008). By continuously reflecting on their own perception and emerging understanding, the research team was compelled to problematize and reflect on taken-for-granted assumptions to let the phenomenon in question show itself more fully. The understanding process was thereby slowed down to let surprising and new meanings arise that otherwise might have been veiled by the researchers’ personal perceptions or by established meanings of the phenomenon.

The researchers first sought to grasp of the whole data set by thoroughly reading all the transcripts. Keywords were marked, and notes were made in the margins. A narrative report was written for every interview that every member of the research group was part of, for validation. Thereafter, the identification focused on the recognition of meaningful parts or units. The texts were divided into fractions of meaning, and for each separate interview, significant meanings were identified and grouped into larger units of meaning. When all meaning units were identified, the researchers formed clusters of meaning by merging those that belonged together. In this way, a temporary understanding of patterns of meaning arose. All clusters were interrelated and written in a synthesis with the purpose of identifying the essential meaning of the phenomenon. These moves were made commonly by all five authors and through several reflection sessions, and until agreement on the meaning of the findings was considered satisfactory. Finally, to verify the outcome, a peer audit (a forum for fellow researchers) was established to discuss the outcome of the study.

When presenting the findings below, the essence of the phenomenon is first described followed by its further constituents, which are the particulars of the general structure of the phenomenon being studied. The rational here is that the particular constituents, which display the more contextual themes of meaning, must be understood as figures against the essential meanings as background (K. Dahlberg et al., 2008). The essential meanings together with the constituents form a general structure of meanings for the phenomenon being studied. Within this structure, there are no strict boarders between the themes of meaning.

## Findings

### Essence

The essential meaning of the phenomenon, initial breastfeeding complications as they are lived and experienced by mothers and how these mothers can be supported, can be described as situated on “the fringes of life,” meaning that one is going through existential extremities. Concerns include former and present expectations of the new situation, an experience where so much is at stake that it may dislocate the situation, experiencing severe nourishment complications, and finding solutions to make the breastfeeding situation work. Experiencing existential extremities is expressed in terms of “Expectations fulfilled and expectations coming to naught” is expressed as an initial and overall great love for an infant that enters one’s life and at the same time a deep sadness and despair regarding the confrontations with oneself related to severe challenges concerning complicated nourishing situations that arise. This is an incomparable vulnerability that relates to a new and trembling openness of becoming a responsible parent that comes as an initial shock-like confrontation in a situation where so much is at stake.

The phenomenon means movements between expectations from oneself and others in being and in becoming a “fulfilled” mother, thus managing both the new role and the feelings of failure and despair when not being capable of nursing/feeding or satisfying the child. This is given expressions like wavering between powerlessness and joy. The close ties envisioned during pregnancy are put at stake regarding expectations of the managing role of being a nourishing mother and the challenges related to nursing and feeding the child in the complex breastfeeding situations described because expectations are both fulfilled and come to naught. Complications arise when the infant refuses to take the breast, when it hurts too much because of functional difficulties, or when the child cannot retain the milk because of anatomic disabilities.

The mothers are seeking advice and support in finding a situation that can work to finding solutions through resistance as an existential extremity. They are relentlessly walking the uphill struggle to finding solutions. A vulnerability becomes prominent in the continuous striving to breastfeed or to give it up; there are no apparent solutions, nothing to cling on to, like a constant wavering between powerlessness and joy. There is relief when solutions are found and care is offered and given, but there is also grief and despair at the discovery of physiological hindrances that would have been possible to avoid or adjust at an earlier stage if they had been seen and confirmed. An increased belief in themselves becomes present when looking back, thus strengthening the sense of motherhood and the insights that grow with the struggle. Gaining confidence, they can move on.

The significance of the lived experience in nourishing an infant having severe breastfeeding complications can be further understood by its constituents as described in the following.

### Expectations Fulfilled and Expectations Coming to Naught

An interwoven existentiality between mother and infant exists, beginning during the 9 months of pregnancy when the infant is carried in the mother’s womb. This time leads to expectations and thoughts of the new life with the new baby. The participants describe the importance of being able to breastfeed their child, and moreover, it becomes one of the deepest meanings of life itself and is given expressions such as being the ultimate love that one could never have imagined would occur in their life in this way.

The love of this child is limitless. . . actually! I have a husband and siblings who have children and whom I love boundlessly, but then this child comes with a further and new dimension I never felt before.

Along with feelings of limitless love and euphoric experiences of the expanding connection to the new life, the first encounters with the infant can come as a shock when overwhelming feelings of responsibility occur. When severe unexpected challenges to nourishing and nursing the infant arise, being able to interpret or understand oneself in a situation that is completely new becomes an existential turning point. It is important to get hold of situations that now claim the whole of one’s existence and that are nonnegotiable and for which there are no written methods. No one can tell you in advance what to do or what must be done that is consistent with the actual situations that occur. Much is at stake in such situations, and life becomes vulnerable.

Everything is supposed to be “a bunch of high spots” . . . and you are there in the midst of it all and realize it is your responsibility—we have to solve this, and we don’t have the faintest idea how.

Simultaneously, a taken for grantedness to be able to breastfeed the infant is there, and it is expected by her surroundings that the mother will breastfeed her infant. Friends, relatives, and health care personnel initially and always ask questions related to the breastfeeding situation, not to all other matters concerning parenthood and the infant.

## Wavering Between Powerlessness and Joy

The mothers had heard people talk about their unconditional love for their newborns. The joy of motherhood and breastfeeding are characterized as being blessed moments, and as such when feeding works, it gives a profound feeling of both joy and freedom that affects the experience of motherhood—a successful feeling of mastering this new role. When initial complications arise and expectations for a lovely, joyful, and successful breastfeeding are not met, feelings of powerlessness, failure, despair, and sorrow occur.

Breastfeeding is strongly associated with one’s identity as a mother, and initial breastfeeding difficulties can make the infant totally ignore or refuse the breast. These situations not only complicate previous intentions to breastfeed the infant but also cause wounds to the identity as a mother and in some cases causing distress and anger toward the infant.

Nevertheless, the expectation of unconditional love fails, as one of the participants stated:

. . . it was that first initial time I had heard them describe this as instant love . . . and then why could I not feel in this way. . .it was really difficult taking care of that child and trying to survive. . .

When initial complications become a fact and expectations for a lovely, joyful, and successful breastfeeding are not met, feelings of powerlessness, failure, despair, and sorrow occur.

I really wanted to breastfeed my child, but he was constantly hungry for 2 months—which ended up being because of a short tongue—and he was constantly very upset and distressed, and then he had problems with sleeping and woke up every 45 minutes and I could not sleep. After 6 weeks I collapsed and could not manage to breastfeed anymore—he struggled against it—I felt bad because this was not the way it should be. And I did not like him and that was very tough because it was not his fault.

Furthermore, it is a sensitive area when friends and relatives elaborate on the breastfeeding situation and the nourishment problems. When telling about their complications, the mothers feel that their concerns are diminished, like suspicions of suckling positions, sleeping disorders, feeding frequencies, and vomiting in terms of volume and frequencies. When explaining their concerns about the infant having different ways of gripping the nipple that would cause severe pain and create soreness and bruises, they felt that health care personnel tried to normalize these problems. Sometimes they had to return 2 or 3 times over a period of 6 weeks before being confirmed that the infant was suffering from pyloric stenosis or a too tight lingual frenum that needed to be operated on or cut in order for them to get a better grip. This period is a burdensome time because so much is at stake for the mothers.

When finding a functioning solution, the breast or bottle-feeding situations are described as intimate and sensitive experiences and as a time of deepened presence with the infant. They are characterized as being blessed moments, and as such when feeding works, it gives a profound feeling of both joy and freedom that affects the experience of motherhood—a successful feeling of mastering this new role.

One mother stubbornly continued to breastfeed, despite the severe soreness of her nipples, and then she finally realized she was suffering from a syndrome making her milk coagulate that required soya lecithin to be able to breastfeed her infant. This might even lead to giving up the breastfeeding completely.

I got these extreme sores that would not heal, and I was only crying when breastfeeding. And she (the child) took a severe grip and was holding with her lips hard around it instead of getting a so-called duck lip . . . so I had to bend out the lips..and this gave rise to mastitis infections and I had to seek help . . . I knew it was wrong—the position and all.

Finding out that the infant has physical disorders that complicated the breastfeeding situation gave confirmation to the suspicions or notions that something was wrong.

When T (the infant) was 3 months, she had surgery for the pyloric stenosis that she was born with. I fought this time with trying to feed her, and she vomited extremely often, and I gave her milk all the time—and time and again this was not paid attention to as I confronted the healthcare personnel . . . and I felt that no one took me seriously and she waited 3 months and vomited about hundred times a day and no one questioned it . . . so it was a real fight to get through these first 4 months . . .

Having to fight to solve the breastfeeding problems contributed to a despairing sense of not being heard or listened to, of not being taken seriously. This creates anger toward the health care personnel because their concerns are not being confirmed. The breastfeeding situations might be solved if listened to, and thus, health care personnel need to relate individually and adjust to every situation—and not normalize or belittle the situation.

## Finding Solutions Through Resistance

There is a continuous seeking of sources to elaborate on and get answers to challenging and complicated situations. Friends, social media, relatives, health care personnel, and partners are approached. It is important to choose the “right” kind of social media to avoid being confronted with unwanted things or to avoid being overloaded with information, thus creating potential problems of uneasiness and vulnerability. At the same time, powerful support is given from their surroundings when the breastfeeding situation does not work. Older female relatives give their contributions and advice as well as friends and colleagues.

Insecurity and a feeling that something is wrong initiates visits to health care centers, and health care encounters are described as both confirming and supporting as well as not being individualized. Overly simple advice such as “Keep on fighting” as a mantra creates feelings of loneliness and detachment in the challenging situations. It is not permitted to express failure. However, being confirmed in the assumptions that something is at stake is a relief.

Finding creative solutions is preceded by several considerations, and there are no easy decisions to make. This represents various forms of processing and deals with giving up on expectations and intentions to breastfeed, which had become a strong intention and struggle over time.

To finally do it—to give up breastfeeding—can lead to both relief and sorrow. Feelings of sorrow occur when the decision to stop is not decided by the mother but by the infant who fails in taking the breast. Sorrow and grief in the process occur because of intentions that are not possible to accommodate—being willing and not willing to breastfeed the infant. Implicitly, the process of stopping the effort of breastfeeding also includes a feeling of becoming independent again and a relief in having made a clear decision.

So, after all, I realized after a while that I had taken breastfeeding for granted. This was important to me, and then a lot of people said: “Why don’t you just give up, you don’t have to breastfeed, let go of the prestige!”

Giving up breastfeeding and instead bottle-feeding the infant could generate insights of a new kind, for example, the realization that to bottle-feed in public could even represent feelings of guilt. This is because this is understood as not being able to breastfeed and again is a symbol of not being a successful mother. To deal with others’ prejudice and one’s own shortcomings is experienced as distressing and worrying. When finding a functioning solution, the breast or bottle-feeding situations are described as intimate and sensitive experiences and as a time of deepened presence with the infant.

The struggle with the intentions of mastering the situation leads to an increased belief in themselves and strengthens their motherhood.

To believe in myself—my own sense and knowledge of the child and situation instead of listening to others . . . I know now that it could have gone better at an earlier stage if I had been more secure from the start . . .

A wish to get help to make the situation of nourishing the infant function in better ways is an essential longing, or to get a confirmation on a decision to terminate breastfeeding—to end the struggle—and a wish to get help to rely on themselves in a challenging and new situation.

## Discussion

Our findings confirm the importance and necessity of listening to and acknowledging mothers in their breastfeeding situations, which has been seen in previous studies (Palmér, 2019; Palmér et al., 2015). Furthermore, our findings also highlight the importance of continuous and tailor-made professional breastfeeding support, which is also in line with earlier studies (Renfrew et al., 2012). Nonetheless, in contrast to these previous findings, we also found that despite their contacts with professional units for breastfeeding support, the mothers still did not feel listened to, and they continued to struggle. Similarly, our results emphasize how existential insecurity makes the encounter between mothers and health care personnel intrinsically difficult. Due to these inherent existential insecurities in the encounters between health care personnel and mothers, rigid methods and descriptive information dictating the conditions of such meetings will hardly create the necessary circumstances for a beneficial and caring encounter. Rather, it becomes evident that the health care personnel should allow space for the “fringes on life”—the expressions of extremities experienced by the mothers—to properly recognize and provide for their individual needs.

The findings also highlight the importance of the health care personnel’s professional knowledge, which is demanded and of necessity for the mothers in their situations. As was observed here, health care personnel sometimes fail in identifying instances of shortened tongue, coagulating breast milk, or pyloric stenosis. While professional knowledge related to the physiological aspects of breastfeeding and variations of complications at a deeper level is therefore a requirement of health care personnel, it is nonetheless crucial that they also be aware of the limits of professional knowledge. The mothers’ own insights into their needs and their conditions must be respected, and this insight must be considered to strengthen mothers’ self-trust.

The most professional relationship is always a personal one, as the professional and the personal are intertwined in the mediation of caring (Holopainen et al., 2019). We suggest that by applying a lifeworld-led caring approach, misunderstandings and frustrations like the mothers experienced here may be avoided. According to lifeworld-led caring (H. Dahlberg et al., 2019; Todres et al., 2006), caring cannot be a one-way act where nurses provide overall information and advice. This is not, and is never likely to become, proper caring. Instead, in the context of a phenomenological approach in which one’s own understanding is constantly bridled and reflected upon, caring be analogous to a research project, dealing with aspects of qualities and the sensing of subtle and often undefined spaces rather than dealing with absolutes and predefined certainties. Thus, breastfeeding mothers at differing stages of extremities but sharing a similar state of insecurity can be properly met, understood, and cared for not through a method in the sense of information to follow, but as an encounter where the mothers’ individual stories and situations guide the care that is given. By conveying a lifeworld-led caring perspective (i.e., H. Dahlberg et al., 2019; Todres et al., 2006), we suggest that some of the barriers between the need for individualized care and the health care personnel can be overcome.

In our study, we found that mothers experienced that they were listened to but not heard in their new and challenging situation, indicating a blunted “clinical sensitivity” on the part of the health care personnel. In contrast to professional knowledge, clinical sensitivity is the mental activity of remaining open to otherness in a caring situation, and this forms the ability to be present, open, and listening (Wiklund Gustin, 2018). Gadamer (2013) was one of the first to describe the dangers of automated or method-based relationships concerning human conditions. Believing that we know something about the other may abruptly end the intersubjective elaboration and thus give significance to an encounter, as opposed to entering into it with a tenet that we do not know (i.e., bridling our understanding) and that we remain open to the otherness—that something can be perceived differently by the other. Therefore, although it would perhaps be tempting to seek to ameliorate a blunted clinical sensitivity by developing new methodological cures in the form of routines and protocols to follow in the caring situation, this is precisely what should be avoided. If you, like an informed biologist, already know what the patient is about to express, chances are that you will only have your preconceptions confirmed. Instead, clinical sensitivity is in many ways aesthetic knowledge (Chinn & Kramer, 2017); it gives rise to a concentration and a presence in the room or in the encounter that may lead to a change of horizon. Thus, by training forms of aesthetic knowledge, the health care personnel’s clinical sensibility can be developed, and the encounter can hopefully be experienced as a genuinely caring one in which patients are both listened to, heard *and* understood (Bullington et al., 2019; Holopainen et al., 2019). Whether developing such clinical sensibility is possible at all within an educational framework that is mainly dedicated to promoting clinical *knowledge* and to conveying this information is an important question but is beyond the scope of the present work.

To be in a situation of existential insecurity as experienced and voiced by the breastfeeding mothers in our study is to be in the fringes on life. The intention to be a good breastfeeding mother is confronted by a multitude of challenges, and no fixed point of certainty is given. This constantly being in extremity poses a unique difficulty for both mothers and health care personnel because no consistent answers or methods exist to counter it, and indeed cannot exist. Still, by acknowledging the vulnerability of the situation, and the lack of recipes for a quick fix, health care personnel could instead be guided by the openness of a clinical awareness that questions are sometimes more important than answers in the encounter between humans, in that this can yield both listening *and* understanding.

## Strengths and Limitations

All researchers in this study are teaching specialist programs in health care, and we were confronted by the lack of knowledge of maternal breastfeeding complications that exists within primary health care in Sweden. This has led us to introduce in-depth teaching in our programs concerning these matters.

The participating mothers showed great interest in the study, and we found that the study was relevant for the participants based on their wish to express the importance of developing a better understanding of their situations. They showed us the importance of bringing their voices into research because of their substantial experiences. The understanding of participation generated by this study cannot claim to capture the full complexity of what mothers with breastfeeding complications experience as significant. Our findings are based on 12 interviews and might be further explored or modified.

This study’s transferability must be understood in terms of what the results of this study mean to the women having severe breastfeeding challenges. By describing a phenomenon in detail, one can begin to evaluate the extent to which the conclusions drawn are transferable to other times, settings, situations, and people. Rich and thick descriptions were included in this article showing verbatim quotes from the informants (Morse, 2015). The study was carried out within a primary health care context in Sweden, and as such, the results cannot immediately be transferred to contexts with different health care systems and laws. However, the essence and the constituents in this study could have relevance for other mothers in the same situations. The working process has been going on over a period of 12 months, with all five authors involved in each step as a validity marker. Originality can be identified because the study is unique in being an empirical study within a primary health care context that focuses on the lived experiences of mothers having severe breastfeeding complications, which is an underexplored area. The usefulness and applicability (Morse, 2015) of the study may be seen in the future as it offers an understanding of the existential challenges related to breastfeeding complications and the importance of giving individualized care.

Given the outcomes of the study, the researchers have initiated changes in the health care system within the region of Stockholm. Several pediatric nurse-led breastfeeding clinics have been realized in the last 6 months. The experienced shortcomings in knowledge considering breastfeeding complications have been noticed and considered in both pediatric nurse and district nurse programs. Furthermore, an extended specialist education for pediatric nurses and district nurses (primary health care nurses) has also been initiated.

## Conclusion

The lived experiences involved in initial breastfeeding challenges and complications must be identified and understood because they are known to have an impact on a mother’s decision to breastfeed or not, which in turn may affect the child’s health and wellbeing. Reflective lifeworld research approach seems to be an appropriate tool for developing and contributing to this deeper understanding because it sheds lights on the phenomenon under study, in this case, initial breastfeeding complications as lived and experienced by mothers struggling with nourishing their infant. In addition, the approach may have potential for assisting professionals in identifying appropriate knowledge and understanding to provide mothers with adequate care and support, so that they can be both listened to and heard. We suggest that lifeworld-led caring can be a fruitful approach in health care situations overall.

## References

[bibr1-10497323211002484] Adams C. van Manen M. A. (2017). Teaching phenomenological research and writing. Qualitative Health Research, 27(6), 780–791. 10.1177/1049732317698960 28682719

[bibr2-10497323211002484] Almqvist-Tangen G. Bergman S. Dahlgren J. Rosvall J. Alm B. (2012). Factors associated with discontinuation of breastfeeding before 1 month of age. Acta Pædiatrica, 101(1), 55–60. 10.1111/j.1651-2227.2011.02405.x 21767302

[bibr3-10497323211002484] Brockway M. Venturato L. (2016). Breastfeeding beyond infancy: A concept analysis. Journal of Advanced Nursing, 72(9), 2003–2015. 10.1111/jan.13000 27189839

[bibr4-10497323211002484] Bullington J. Söderlund M. Bos Sparén E. Knäck Å. Omerov P. Cronqvist A. (2019). Communication skills in nursing: A phenomenologically based communication training approach. Nurse Education in Practice, 39, 136–141. 10.1016/j.nepr.2019.08.011 31487674

[bibr5-10497323211002484] Cast A. D. (2004). Well-being and the transition to parenthood: An identity theory approach. Sociological Perspective, 547(1), 55–78. 10.1525/sop.2004.47.1.55

[bibr6-10497323211002484] Chasson M. Taubman-Ben-Ari O. (2020). Early maternal caregiving: A conceptualization of maternal compassion preoccupation. Qualitative Health Research, 30(9), 1303–1313. 10.1177/1049732320908570 32111143

[bibr7-10497323211002484] Chinn P. Kramer M. K. (2017). Knowledge development in nursing (10th ed.). Elsevier.

[bibr8-10497323211002484] Dahlberg H. Dahlberg K. (2003). To not make definite what is indefinite: A phenomenological analysis of perception and its epistemological consequences in human science research. The Humanistic Psychologist, 31(4), 34–50. 10.1080/08873267.2003.9986933

[bibr9-10497323211002484] Dahlberg H. Ellingsen S. Martinsen B. Rosberg S. (2019). Phenomenology in practise-research in a Scandinavian perspective (1st ed.). Liber.

[bibr10-10497323211002484] Dahlberg K. Dahlberg H. Nyström M. (2008). Reflective lifeworld research (2nd ed.). Studentlitteratur.

[bibr11-10497323211002484] Deave T. Johnson D. Ingram J. (2008). Transition to parenthood: The needs of parents in pregnancy and early parenthood. BMC Pregnancy Childbirth, 8, Article 30. 10.1186/1471-2393-8-30 PMC2519055 18664251

[bibr12-10497323211002484] Gadamer H. G. (2013). Truth and method. Bloomsbury Academic.

[bibr13-10497323211002484] Hauck Y. L. Blixt I. Hildingsson I. Gallagher L. Rubertsson C. Thomson B. Lewis L. (2016). Australian, Irish and Swedish women’s perceptions of what assisted them to breastfeed for six months: Exploratory design using critical incident technique. BMC Public Health, 10, Article 1067. 10.1186/s12889-016-3740-3 PMC5057437 27724932

[bibr14-10497323211002484] Holopainen G. Nyström L. Kasen A. (2019). The caring encounter in nursing. Nursing Ethics, 26(1), 7–16. 10.1177/0969733016687161 28095761

[bibr15-10497323211002484] Husserl E. (1980). The idea of phenomenology. Kluwer Academic Publishers.

[bibr16-10497323211002484] Kvale S. Brinkman S. (2014). Qualitative research interviewing (3rd ed.). Studentlitteratur.

[bibr17-10497323211002484] Larsen-Schilling J. Kronborg H. (2013). When breastfeeding is unsuccessful—Mothers’ experiences after giving up breastfeeding. Scandinavian Journal of Caring Sciences, 27(4), 848–856. 10.1111/j.1471-6712.2012.01091.x 23057626

[bibr18-10497323211002484] Lawrence E. Nylén K. Cobb R. J. (2007). Prenatal expectations and marital satisfaction over the transition to parenthood. Journal of Family Psychology, 21(2), 155–164. 10.1037/0893-3200.21.2.155 17605538

[bibr19-10497323211002484] Lessen R. Kavanagh K. (2015). Position of the academy of nutrition and dietetics: Promoting and supporting breastfeeding. Journal of the Academy of Nutrition and Dietetics, 115(3), 444–449. 10.1016/j.jand.2014.12.014 25721389

[bibr20-10497323211002484] Morse J. M. (2015). Critical analysis strategies for determining rigor in Qualitative Inquiry. Qualitative Health Research, 25(9), 1212–1222. 10.1177/1049732315588501 26184336

[bibr21-10497323211002484] National Board of Health and Welfare. (2017). Statistics on breastfeeding 2017. https://www.socialstyrelsen.se/en/search-results/?q=breastfeeding&_t_dtq=true

[bibr22-10497323211002484] The National Handbook of Child Health Services. (2019). Breastfeeding guidance. https://www.rikshandboken-bhv.se/amning-och-nutrition/amningsvagledning/

[bibr23-10497323211002484] Palmér L. (2019). Previous breastfeeding difficulties: An existential breastfeeding trauma with two intertwined pathways for future breastfeeding—Fear and longing. International Journal of Qualitative Studies on Health and Well-Being, 14(1), Article 1588034. 10.1080/17482631.2019.1588034 PMC6442107 30893016

[bibr24-10497323211002484] Palmér L. Carlsson G. Brunt D. Nyström M. (2015). Existential security is a necessary condition for continued breastfeeding despite severe initial difficulties: A lifeworld hermeneutical study. International Breastfeeding Journal, 10(1), 17. https://10.1186/s13006-015-0042-9 25960763 10.1186/s13006-015-0042-9 PMC4425864

[bibr25-10497323211002484] Region Stockholm. (2019). Annual report: Child health care in Stockholm county 2019. Child Health Care Unit. https://vardgivarguiden.se/globalassets/kunskapsstod/bvc/bhv-rapporter/arsrapport-barnhalsovard-2019.pdf

[bibr26-10497323211002484] Region Stockholm. (2020). Basic program: Care during pregnancy. Antenatal Health Care Unite. https://vardgivarguiden.se/globalassets/kunskapsstod/bmm/basprogram.pdf

[bibr27-10497323211002484] Renfrew M. McCormick F. M. Wade A. Quinn B. Dowswell T. (2012). Support for healthy breastfeeding mothers with healthy term babies. Cochrane Library, 5, CD001141.pub5. 10.1002/14651858 PMC3966266 22592675

[bibr28-10497323211002484] Ryan K. Todres L. Alexander J. (2011). Calling, permission, and fulfillment: The interembodied experience of breastfeeding. Qualitative Health Research, 21(6), 731–742. 10.1177/1049732310392591 21139042

[bibr29-10497323211002484] Schafer E.-J. Campo S. Colaizy T.-T. Mulder P.-J. Breheny P. Ashida S. (2016). First-time mother’s breast-feeding maintenance: Role of experiences and changes in maternal perceptions. Public Health Nutrition, 20(17), 3099–3108. 10.1017/S136898001700221X PMC10261612 28879823

[bibr30-10497323211002484] Swedish Food Agency. (2012). Good food for infants under one year. https://www.livsmedelsverket.se/globalassets/publikationsdatabas/andra-sprak/bra-mat-for-spadbarn/good-food-for-infants-under-one-year-livsmedelsverket.pdf

[bibr31-10497323211002484] Todres L. Galvin K. Dahlberg K. (2006). Lifeworld-led healthcare: Revisiting a humanizing philosophy that integrates emerging trends. Medicine, Health Care and Philosophy, 10(1), 53–63. 10.1007/s11019-006-9012-8 16847724

[bibr32-10497323211002484] van Manen M. (2014). Phenomenology of practice: Meaning-giving methods in phenomenological research and writing. Left Coast Press.

[bibr33-10497323211002484] Victora C. G. Bahl R. Barros A. J. D. Franca G. V. A. Horton A. Krasevec J. Murch S. Sankar M. J. Walker N. Rollins N. C. (2016). Breastfeeding in the 21st century: Epidemiology, mechanisms, and lifelong effect. The Lancet, 387(10017), 475–490. 10.1016/S0140-6736(15)01024-7 26869575

[bibr34-10497323211002484] Wiklund Gustin L. (2018). Being mindful as a phenomenological attitude. Journal of Holistic Nursing, 36(3), 272–281. 10.1177/0898010117724928 28793814

[bibr35-10497323211002484] Williamson I. Leeming D. Lyttle S. Johnson S. (2012). “It should be the most natural thing in the world”: Exploring first-time mothers’ breastfeeding difficulties in the UK using audio-diaries and interviews. Maternal & Child Nutrition, 8, 434–447. 10.1111/j.1740-8709.2011.00328.x 21696542 PMC6860601

[bibr36-10497323211002484] World Health Organization. (2011). Exclusive breastfeeding for six months best for babies everywhere. https://www.who.int/mediacentre/news/statements/2011/breastfeeding_20110115/en/

[bibr37-10497323211002484] World Health Organization. (2020). Infant and young child feeding. https://www.who.int/news-room/fact-sheets/detail/infant-and-young-child-feeding

[bibr38-10497323211002484] World Medical Association. (2018). Declaration of Helsinki: Ethical principles for medical research involving human subjects. https://www.wma.net/policies-post/wma-declaration-of-helsinki-ethical-principles-for-medical-research-involving-human-subjects/19886379

